# Future Challenges in the Assessment of Proprioception in Exercise Sciences: Is Imitation an Alternative?

**DOI:** 10.3389/fnhum.2021.664667

**Published:** 2021-06-02

**Authors:** Jesús Munóz-Jiménez, Daniel Rojas-Valverde, Kiko Leon

**Affiliations:** ^1^Facultad Ciencias del Deporte, Universidad de Extremadura, Cáceres, Spain; ^2^Centro de Investigación y Diagnóstico en Salud y Deporte (CIDISAD), Escuela Ciencias del Movimiento Humano y Calidad de Vida (CIEMHCAVI), Universidad Nacional, Heredia, Costa Rica

**Keywords:** visuomotor, assessing, test, sensorimotor, somatosensory

## Introduction

To perform any body movement, a series of sensory processes are required that provide the necessary information. Once this sensory data is integrated at the cortical level, an optimal response is given considering but not limited to space and time. This neuromotor coordination results in a synergic, intentional, and synchronic action. Voluntary movements require a coordinated and efficient muscle contraction mediated by the central nervous system integration processes (Tuthill and Azim, 2018). To do so, the cerebellum and brain regulate the sensitive data obtained from a multi-model sensory system. In the case of corporeal control or body control, it is required information from the somatosensory sense as the one that gives information from the “inside” of the body (ten Donkelaar et al., 2020). Once the body interacts with the environment in time and space, this body control needs the ocular, vestibular, tact, and auditory exteroceptive senses to obtain information from the context. This is how the term spatio-temporal body control could be used.

The somatosensory sense involves thermoception, nociception, equilibrioception, mechanoreception, and proprioception. This last sense allows humans to detect the position and motion of musculoskeletal structures (e.g., muscle [muscle spindle, Golgi tendon organs, palisade endings] and joint [Ruffini endings, Pacinian corpuscles], ligament) in relation with each other and space (Blumer, 2010), but also provides force generation sensation that allows regulating force output (Lin et al., 2014), kinesthesia, and a sense of change in velocity. It also includes awareness of the body in space (Norris, 2011), necessary for an appropriate spatial and temporal limb coordination during movement (Corkery and Iversen, 2016).

Our brain's capability to make a representation of our body is at least quite precocious (Berlucchi and Aglioti, 1997) and becomes active during observation of another individual's actions and self-execution of a movement; consider that newborns are sensitive to others' expressions and body movements as a way to interact with others (Farroni et al., 2007). Somatosensory inputs, especially from proprioceptors, are undoubtedly essential for developing body schema, body image, and body awareness (Berlucchi and Aglioti, 1997). In human exercise sciences, proprioception is crucial to achieve an operational goal (efficacy; e.g., sports performance) and minimize the biomechanical demands of a specific task (efficiency, e.g., optimize energy expenditure and avoid injuries; Ogard, 2011; Liutsko, 2013; Maurice et al., 2019). It is understood as the ability to link sensory signals from mechanoreceptors to detect corporeal segments' position and movement concerning space (Han et al., 2016). This definition considers that proprioception should not be understood as a physiological property but the integration of physiological and psychological functions.

Han et al. (2016) have explained the physiological and psychological role of hardware and software interaction. The first perceives and provides proprioceptive data from and to the central nervous system, and the second integrates this information and develops a response. This means that there is a permanent feedback and feedforward loop and interaction in this physiological and psychological autonomous network (Corr, 2010). Since 1863, this interaction between movement and other exteroceptive sense interaction has been defined as muscle sense (Liutsko, 2013). Some recent literature has suggested that proprioception differs by individual differences as individual construct, experiences, personality, sex, age, and others development and situational dependent factors (Liutsko, 2013; Liutsko et al., 2014, 2018; Tous Ral and Liutsko, 2014).

Improving movement and spatiotemporal body control is required to train and obtain permanent feedback. The difficulty in improving body control and underlying movements lies in detecting suitable modifications, leading to performance enhancement (Maurice et al., 2019). In this sense, a series of evaluation methods have been proposed to assess proprioception. The main assessing techniques for proprioception have differentiated between two main system functions: detecting the static position and detecting basic movement. Among the principal evaluations is (a) threshold to detection of passive motion, (b) joint position reproduction or matching, (c) active movement extent discrimination assessment (Lephart et al., 2002; Weerakkody et al., 2008; Han et al., 2016). These evaluations are widely accepted by the international scientific community in sports medicine and rehabilitation and other sport-related research areas. Still, these methods exclude the somatosensory system's interaction with the environment as considered in the definitions mentioned above of proprioception and spatio-temporal body control (Hillier et al., 2015; Ager et al., 2017).

The evaluation of body control and proprioception is limited to relatively simple tests, commonly passive, and differs from the movement's complexity and gesture during physical exercise (Hillier et al., 2015). These proposed methods evaluate the body control in a segmented way and do not consider the highly dynamic nature of specific movements and their interaction with the environment, and usually explore only isolated features (Hillier et al., 2015). An increasing number of researchers in sports and exercise sciences recognize central processing's critical role in proprioception (Han et al., 2016). Considering that during exercise, proprioceptive information is feedback and integrated with other exteroceptors' senses as ocular, vestibular (balance and equilibrium), tact, and auditory, the isolated and segmented evaluation of mechanical movements is not necessarily accurate when applied to complex activities such as actions related to exercise. This is because when moving under predictable circumstances, the proprioceptive feedback modulates the magnitude and timing of muscle activity without major problems, but in most situations during exercise, humans must adapt their movements to the natural environment (Tuthill and Azim, 2018). This indicates that considering the actual assessing techniques, a positive proprioceptive test result under isolated or segmentary conditions is not necessarily practically significant or applicable in real settings.

Therefore, evaluating proprioception methods is necessary to collect objective, valid, and reliable information, but that considers the limitations and natural challenges of moving in a real exercise scene. The proprioceptive assessment methods are usually based on detecting positions previously performed, using copying or position matching tasks (Hillier et al., 2015). These tasks could be passively or actively performed. Considering the multiple constructs endorsed to proprioception, such tests usually raise more doubts than answers to a practical problem; the main concern has been the validity of these tests in daily activities and how applicable these measures are to exercise sciences. Consequently, considering the holistic definition of proprioception and its role in body control, this manuscript aims to identify knowledge gaps in the assessment of proprioception and explore the potential of imitation to assess it in a global manner to increase its practical significance.

## Future Challenges in the Proprioception Assessment

Considering the serious questions regarding the practical significance of the tests currently performed to assess proprioception, there is a need to develop tests and testing batteries that consider other exteroceptive senses' influence on proprioceptive integration future motor response during spatiotemporal body control (Ogard, 2011). In this sense, there should be a consideration of some situational and contextual variables that usually interfere with the proper proprioceptive system functionality during typical sport and exercise execution such as fatigue, multi-joint actions, and positioning, different exercise intensities execution, psychological stress, attention, injuries, emotion, among others.

It should be investigated how some clinical exercise-related issues affect the proprioceptive data and how the suppression of other exteroceptive senses could influence the decremental or enhancement response to a specific stimulus. In this sense, more detailed information is needed regarding how to prescribe, train, and evaluate the subconscious proprioception exercise related tasks, considering the actually available tests are mainly conscious and passive (Ogard, 2011).

More in-depth understanding is needed regarding the physiological and biochemical cascade during the proprioceptive sensory system functionality. Additionally, more information is needed when analyzing how much the exteroceptive senses and somatosensory systems contribute to body control and how they interact to emit an optimal response. It is understood that both peripheral and central nervous responses vary by age, experiences, physical fitness, and other personal and contextual variables. So it is required to better comprehend how these different situational variables could influence proprioceptive and motor responses (Proske and Gandevia, 2012).

Finally, considering there is a discussion of how to train the proprioceptive capabilities, some training methods should be tested using clinical research considering both the conscious and subconscious proprioception properties (Chu, 2017). Besides, the validity, reliability, and practical significance of these tests should be explored and improved. In this sense, hand by hand, with the technological developments in hardware and software, researchers have a wide range of available options that could assess proprioception in an objective manner (Hillier et al., 2015).

In this way, considering the technological advances in imaging, biomechanical, and motor evaluation, it is possible to access objective, reliable, and consistent data of a movement or several movements of the center of mass and upper and lower limbs. Variables such as acceleration, deceleration, movement velocity, motion trajectory, and positioning could be monitored using motion capture techniques, inertial measurement devices, magnetic, angular rate and gravity sensors, and mobile applications. These equipment and systems integrated with proper task protocols allow execution and performance of a complex movement, avoiding the semantic route in which previous knowledge and action meaning could influence basic movement.

## Imitation as an Alternative to Assess Proprioception

Imitation of body movements involves copying tasks or acts without using objects, without leading to an end state, without specific meaning (avoiding semantic route in which previous knowledge and action meaning could influence basic movement), and usually described in terms of body changes postures in space. The ability to imitate body movements is present in humans beginning in the early development stages, involving affective mirroring and copying of body actions with caregivers (Vivanti, 2013).

Imitation ability is supported by a direct visuospatial route in which the visual input resulted in motor output (Vivanti, 2013). Indeed, when a person observes other people's movements, the somatosensory area of the brain that controls motor actions is active (Hanawa et al., 2016; Watanabe et al., 2017). Imitation ability could be assessed via imitation of prerecorded human whole-body complex motions in real time. Contrarily, when a self-execution (both imitator-imitated roles) is mapped and compared with a subsequent imitation attempt, the visual input is limited and somatosensory sense providing the most information and feedback to achieve the repetition of a static or dynamic task.

This proposal is based on a motor imitation model using self-execution meaningless movements as a reference. This model considers three stages of information transformation proposed previously as a perception-motor loop (Rizzolatti et al., 2001; Vogt and Thomaschke, 2007). The first one computes the input signal (self-execution movements) into an internal representation, modulated by attention and context. The second one occurs when the person imagines or intends to execute an imitative act. And the third one occurs when the person executes motor imitation mediated and mapped by body motor schema and proprioception, then translated into motor command executed by musculoskeletal structures (Holmes and Spence, 2004).

Copying and imitating depend on representing the body schema in the posterior parietal cortex and dorsal premotor cortex. This is why the integration of ocular, vestibular (balance and equilibrium), and sensorimotor inputs and especially proprioception is fundamental during these kinds of tasks. This is achieved by computing the set of changes required to move or repose the body-schema representation to match the initial action. In other words, the imitation ability suggests the existence of an internal representation of the body and the relative positioning and motion of body segments (Lopez and Blanke, 2010). This facilitates recognizing and copying static and dynamic postures (Buxbaum et al., 2014; Wong et al., 2018). The latter are hand in hand with the ability to repeat the specific movement trajectory.

Considering the complexity of the human movement during exercise and that proprioception is usually assessed via isolated tasks obviating the spatiotemporal factors of movement, imitation rises as an option to globally evaluate the capacity to detect the musculoskeletal structures positioning concerning space and time. Consequently, imitation allows the measure of proprioception considering other exteroceptive senses information. This assessment alternative acknowledges the vital role of spatio-temporal body control during exercise and human movement.

## Conclusions

Proprioception in exercise sciences is understood as the brain's conscious and unconscious capability to detect musculoskeletal body structures (e.g., muscle, tendon, joint), their position, and movement in relation to space and time. This allows the brain to represent the body, considering the information from somatosensory inputs to regulate body segments' position and execute coordinated movements. Exteroceptive senses feedback these processes (e.g., ocular, vestibular, tact, and auditory) and need neuromotor coordination resulting in a synergic, intentional, and synchronic action.

Considering the complexity of the physiological pathways of the processes underlying proprioceptive sensing and the integrated functions with other exteroceptive senses in real settings, the existing assessment tests explore only isolated features and lack practical significance. Rather than evaluations that consider isolated segmental movements, some proprioceptive tests consider the organic challenges of body control when exercising are needed.

Since imitation is innate and a natural pathway during neuroplasticity and learning, we propose a self-imitation based test due to its potential to assess the somatosensory function needed during proprioception. Due to this type of evaluation's capacity to evaluate the somatosensory and proprioceptive capacity to, in an integrated way, detect the different body segments, their position, and movements to execute a previously performed movement. Using the motion tracking available technology, this type of evaluation would quantify the consistency and congruence in positioning, execution force, movement velocity, acceleration, and motion trajectory between two movements executed subsequently. Finally, imitation could potentially be an option that integrates both somatosensory and exteroceptive senses data to assess body control in exercise science.

## Author Contributions

DR-V, KL, and JM-J contributed to the conception, design of the study idea, key information, topic insights, manuscript revision, critical review, discussed, and approved the submitted version. DR-V wrote the first draft of the manuscript. All authors contributed to the article and approved the submitted version.

## Conflict of Interest

The authors declare that the research was conducted in the absence of any commercial or financial relationships that could be construed as a potential conflict of interest.
